# Systematic Comparison of Aroma Profiles and Tea Polysaccharide In Vitro Antioxidant Activities Among Six Anxi Oolong Tea Cultivars

**DOI:** 10.3390/foods15193406

**Published:** 2026-09-24

**Authors:** Zhong Wang, Zhelin Wan, Fanyu Liu, Changlian Wu, Shuaibo Shao, Yuting Li, Zhendong Zhang, Chengzhe Zhou, Cheng Zhang, Yuqiong Guo

**Affiliations:** 1 Anxi College of Tea Science, College of Horticulture, Fujian Agriculture and Forestry University, Fuzhou 350002, China; 2 Tea Green Cultivation and Processing Collaborative Innovation Center, Fujian Agriculture and Forestry University, Fuzhou 350002, China

**Keywords:** *Camellia sinensis* cultivars, volatile organic compounds, tea polysaccharides, antioxidant activity, molecular docking

## Abstract

Oolong tea is prized for its floral aroma and bioactive tea polysaccharides (TPS), yet the independent contribution of cultivar genetic background to aroma chemistry and TPS functionality remains unclear due to confounding effects from environment and processing. Here, we systematically compared volatile organic compound (VOC) profiles and in vitro antioxidant activities of TPS from six Anxi oolong tea cultivars all grown in the same tea garden and processed under identical Qingxiang oolong tea protocols. Using headspace solid-phase microextraction-gas chromatography-mass spectrometry, 39 VOCs were identified across cultivars. Huangjingui exhibited the highest total VOC content (69.01 μg·g^−1^) and the greatest number of compounds with relative odor activity values (rOAV) over 1. Principal component analysis and hierarchical clustering revealed clear metabolic separation among cultivars, with Daye Wulong displaying the most distinct metabolome. PLS-DA combined with rOAV screening identified 12 key differential volatiles that form the chemical basis of floral aroma. Preliminary in silico molecular docking predicted the strongest binding affinity between nerolidol and OR1A1 (−7.54 kcal/mol). Cultivar-specific high rOAV volatiles were characterized, including (E)-2-octenal and 1-phenyl-2-nitroethane in Tieguanyin, 2-phenylethyl-2-methylbutyrate and cis-3-hexenyl isovalerate in Maoxie, α-ocimene and cis-3-hexenyl benzoate in Daye Wulong, and (E,E,Z)-1,3,5,8-undecatetraene in Meizhan, suggesting their potential as chemical markers for cultivar authentication. Crude TPS yields ranged from 2.76% to 3.99%; Tieguanyin had the highest yield and Meizhan the lowest. TPS from Daye Wulong exhibited the strongest DPPH and ABTS^+^· scavenging capacities, with IC_50_ values of 0.58 and 1.95 mg·mL^−1^, respectively, indicating the highest in vitro antioxidant activity among all tested samples. These results suggest that cultivar genetic background may be a primary determinant of both aroma metabolites and TPS bioactivity, providing a theoretical basis for cultivar selection and quality optimization in oolong tea production.

## 1. Introduction

Tea is one of the most widely consumed health beverages globally, and its market value and consumer acceptance are predominantly determined by two core attributes: distinctive sensory flavor and recognized health benefits [1,2]. Among various tea categories, oolong tea is particularly esteemed in domestic and international markets for its complex and rich floral–fruity aroma characteristics [3,4]. Furthermore, oolong tea is abundant in functional macromolecules such as tea polysaccharides (TPS), which have been demonstrated to possess potential biological activities including in vitro antioxidant capacity, immunomodulation, and gut microbiota regulation, making them a prominent research focus in food science in recent years [5,6,7]. Nevertheless, achieving improvements in both the flavor quality and functionality of oolong tea necessitates a thorough understanding of how cultivar influences chemical composition and overall quality.

The quality of tea, encompassing both the profile of volatile organic compounds (VOCs) responsible for flavor and the structure and content of functional components like TPS, is significantly influenced not only by processing techniques but also decisively by the genetic background of the tea plant cultivar [4,8]. As the birthplace of oolong tea, Anxi County in Fujian Province, China, harbors an exceptionally rich diversity of tea plant germplasm resources. Among these, the “Six Famous Teas of Anxi”—Tieguanyin (*Camellia sinensis* cv. Tieguanyin, TGY), Huangjingui (*C. sinensis* cv. Huangjingui, HJG), Maoxie (*C. sinensis* cv. Maoxie, MX), Benshan (*C. sinensis* cv. Benshan, BSS), Meizhan (*C. sinensis* cv. Meizhan, MZ), and Daye Wulong (*C. sinensis* cv. Daye Wulong, DYL)—are classic materials for oolong tea research due to their distinct aromatic characteristics. These six cultivars not only possess a long cultural history in tea production but also hold significant positions as core varieties in oolong tea manufacturing.

In recent years, substantial progress has been made in scientific investigations into the differences in aroma and functional components among different oolong tea cultivars [2,9]. The aroma profiles of TGY and HJG, for instance, exhibit considerable variation and display pronounced cultivar-specific features [2,10]. Studies based on Fujian oolong teas have demonstrated significant differences among cultivars in sensory dimensions such as floral, fruity, and sweet notes [9,11]. At the level of key aroma-contributing substances, multiple critical volatiles have been identified from Fujian oolong teas, including floral and fruity compounds like linalool, geraniol, and benzaldehyde [9,12]. Omics technologies have provided compelling molecular-level evidence for cultivar-based differences in oolong tea. Genomic studies have revealed that the terpene synthase gene family, associated with terpenoid volatile synthesis, has undergone significant gene expansion, structural variation, and specific high expression in high-aroma cultivars such as HJG, establishing the genetic basis for their floral–fruity characteristics [13,14,15]. Transcriptomic analyses indicate that key genes in the limonene metabolic pathway are significantly upregulated in TGY, showing higher activity compared to BSS [16]. Furthermore, genome-wide association studies based on Fujian tea germplasm resources have found that the content of linalool, an important floral substance, is influenced by different allelic variations of the positive regulator CsERF-like [17]. The transcription factor CsMYB62 has also been identified as a crucial positive regulator of linalool and phenylacetaldehyde synthesis [18]. Regarding polysaccharide differences, the content and free radical scavenging activity of TPS vary among different oolong tea cultivars and fermentation degrees, indicating that both cultivar differences and fermentation treatment significantly affect TPS activity [19]. Further research has also shown significant differences in the in vitro antioxidant activity of TPS among different cultivars [20].

Although previous studies have elucidated the mechanisms by which the genetic background of tea plant cultivars affects aroma and functional components from multiple omics perspectives, including metabolomics, genomics, and transcriptomics, existing research has primarily utilized samples from different origins, harvest years, or processing techniques. This approach fails to effectively eliminate the confounding variables of habitat and processing conditions, thereby hindering the definitive attribution of observed differences in aroma and antioxidant activity solely to the cultivar itself. A systematic comparison of aroma profiles and TPS antioxidant activities among the six core cultivars under strictly controlled identical habitat and processing conditions represents a current knowledge gap.

To address this, the present study employed a unified Southern Fujian Qingxiang (light-flavor) oolong tea processing protocol to systematically analyze the differences in characteristic aroma profiles and in vitro antioxidant activities of TPS among the six core cultivars from Anxi. By combining headspace solid-phase microextraction-gas chromatography-mass spectrometry (HS-SPME-GC-MS) with odor activity value (OAV) analysis for key volatile compounds, and utilizing water extraction and ethanol precipitation for TPS isolation followed by in vitro antioxidant evaluation, this study aims to elucidate the differences in sensory quality and functional components of oolong tea from the perspective of cultivar characteristics, thereby providing a theoretical basis for cultivar optimization and processing improvement of Fujian oolong tea.

## 2. Materials and Methods

### 2.1. Samples and Chemical Reagents

To elucidate the material basis underlying the aroma differences among six oolong tea cultivars, fresh leaves were plucked in May 2024 from a single tea plantation in Huqiu Town, Anxi County, Fujian Province, China, ensuring uniform cultivation conditions. The six representative Southern Fujian oolong tea cultivars—TGY, HJG, MX, BSS, MZ, and DYL—were processed into Qingxiang (light-flavor) oolong tea under identical conditions by an experienced tea master, according to the national standard “Technology Regulations for Oolong Tea Processing” (GB/T 35863-2018 [21]). The specific processing parameters were as follows: solar withering for 30 min with one turning in the middle, followed by indoor withering at 25–28 °C and 60–70% relative humidity for 4.5 h; shaking for three rounds, the first and second shakes each followed by resting for 2 h and 3 h, respectively, with a final extended spreading of 12 h after the third shake; total doing-green lasting approximately 18 h in a shaking room maintained at 20–23 °C and 60–80% relative humidity; fixation at 180–200 °C for 3–5 min; and final drying at 70 °C for 5 h. After undergoing primary processing, sorting, sieving, and winnowing, the finished tea samples were obtained and stored in sealed containers at −20 °C until further analysis (Figure 1A).

The main reagents used in this study were as follows: 1,1-diphenyl-2-picrylhydrazyl (DPPH) and 2,2′-azino-bis(3-ethylbenzothiazoline-6-sulfonic acid) diammonium salt (ABTS) were purchased from Shanghai Yuanye Bio-Technology Co., Ltd. (Shanghai, China). L-Ascorbic acid, potassium persulfate, ferrous sulfate heptahydrate, hydrogen peroxide, salicylic acid, anhydrous ethanol, chloroform, n-butanol, acetone, and diethyl ether were analytical grade products from Sinopharm Chemical Reagent Co., Ltd. (Shanghai, China). The internal standard for aroma analysis, ethyl decanoate (purity > 99%), was purchased from Sigma-Aldrich (Shanghai, China). Ultrapure water was used throughout all experiments.

The n-alkane solution (C7 to C40) was purchased from Anpel-trace Standard Technical Services Co., Ltd. (Shanghai, China). Ethyl decanoate (CAS 110-38-3, purity > 99%) was purchased from Shanghai Aladdin Biochemical Technology Co., Ltd. (Shanghai, China). 1-Octanol (purity > 99.5%) was purchased from Shanghai Macklin Biochemical Co., Ltd. (Shanghai, China). Benzaldehyde (purity ≥ 99.5%) and geranylacetone (purity ≥ 99.9%) were purchased from Xiamen Lvyin Reagent & Glass Instrument Co., Ltd. (Xiamen, China). 1-Octen-3-ol (purity ≥ 98%), benzeneacetaldehyde (purity 95%), linalool (purity 98%), nonanal (purity 96%), methyl salicylate (purity ≥ 98%), decanal (purity 97%), geraniol (purity ≥ 98%), β-ionone (purity 98%) and trans-nerolidol (purity ≥ 95%) were purchased from Beijing Yayuan Biotechnology Co., Ltd. (Beijing, China).

### 2.2. Headspace Solid-Phase Microextraction and Gas Chromatography-Mass Spectrometry Analysis of Volatiles

Volatile organic compounds (VOCs) from tea samples were analyzed using HS-SPME-GC-MS. The method was adapted from previous studies with appropriate modifications [22]. Briefly, 0.5000 g of tea powder was accurately weighed into a 20 mL headspace vial, to which 5.0 mL of saturated sodium chloride solution and 10 μL of ethyl decanoate internal standard solution (10 μg/mL in methanol) were added. The vial was immediately sealed.

An autosampler equipped with a 50/30 μm DVB/CAR/PDMS fiber was used. Prior to extraction, the fiber was conditioned at 250 °C in the injection port for 10 min. Samples were equilibrated at 80 °C for 10 min, after which the fiber was exposed to the headspace for adsorption for 45 min. Following adsorption, the fiber was immediately inserted into the GC injector for desorption at 250 °C for 6 min.

Gas chromatographic separation was performed on a GCMS-QP2020 NX (Shimadzu Corporation, Kyoto, Japan) system equipped with an HP-5MS non-polar capillary column (30 m × 0.25 mm × 0.25 μm, Agilent Technologies, Santa Clara, CA, USA). High-purity helium (99.999%) served as the carrier gas at a constant flow rate of 1.2 mL/min. The oven temperature program was as follows: initial temperature 40 °C held for 3 min, increased to 120 °C at 5 °C/min, then raised to 230 °C at 10 °C/min and held for 5 min. The injection was performed in splitless mode. Mass spectrometry employed electron impact ionization with an electron energy of 70 eV. The ion source temperature was set at 230 °C, the interface temperature at 250 °C, and the mass scan range was *m*/*z* 35–500.

Volatile compounds were identified by comparing their mass spectra with the NIST 20 standard library and confirmed by comparing retention indices with those reported in the relevant literature. Semi-quantification was performed using the internal standard method, and the relative content of each component was calculated (μg·g^−1^). To evaluate the contribution of each component to the overall aroma, the relative odor activity value (rOAV) was calculated based on published aroma threshold data [23]. The ratio of the relative content of a compound to its odor threshold in water was calculated as the rOAV.

### 2.3. Extraction and Yield Calculation of Tea Polysaccharides

Tea polysaccharides were extracted using a water extraction and ethanol precipitation method combined with Sevag reagent for protein removal [7,24]. The procedure was as follows: 25.0 g of tea powder passing through a 40-mesh sieve was accurately weighed and mixed with 200 mL of ultrapure water. The mixture was incubated in a 90 °C water bath for 1 h. The extract was centrifuged at 3000 rpm for 10 min, and the supernatant was collected. Three volumes of anhydrous ethanol were added to the supernatant, and the mixture was allowed to stand at 4 °C overnight. Subsequently, the mixture was centrifuged at 4000 rpm for 20 min, and the precipitated crude TPS were collected.

For further purification, the crude polysaccharide precipitate was dissolved in 100 mL of ultrapure water. An equal volume of Sevag reagent (chloroform:n-butanol = 4:1, *v*/*v*) was added, and the mixture was vortexed for 2 min, then centrifuged at 3000 rpm for 10 min to remove proteins. This step was repeated until no visible precipitate appeared at the interface. The supernatant was collected, and three volumes of anhydrous ethanol were again added for re-precipitation. After centrifugation, the resulting precipitate was washed sequentially with 50 mL each of anhydrous ethanol, acetone, and diethyl ether. Finally, the precipitate was dried in an oven at 60 °C to constant weight to obtain crude TPS preparations.

The crude TPS yield (Y, %) was calculated using the following formula:

Y (%) = (M_1_/M_0_) × 100, (1)

where M_1_ is the dry weight of crude TPS preparations, and M_0_ is the dry weight of the tea sample.

### 2.4. In Vitro Antioxidant Activity Assays of Tea Polysaccharides

#### 2.4.1. DPPH Radical Scavenging Activity Assay

The DPPH radical scavenging assay was performed according to previously described methods with appropriate modifications [25]. TPS samples were prepared as aqueous solutions at concentrations of 0.2, 0.8, 1.4, and 2.0 mg/mL. For each measurement, 1 mL of sample solution was mixed with 3 mL of 0.1 mmol/L DPPH-ethanol solution. The mixture was vortexed and incubated in the dark at room temperature for 30 min. Absorbance was measured at 517 nm (A_sample). A sample blank control (A_control) consisted of 1 mL of sample solution mixed with 3 mL of anhydrous ethanol. A blank control (A_blank) consisted of 1 mL of ultrapure water mixed with 3 mL of DPPH solution. L-Ascorbic acid (Vc) solution at equivalent concentrations served as a positive control. The DPPH radical scavenging rate (I, %) was calculated as follows:

I (%) = [1 − (A_sample − A_control)/A_blank] × 100.(2)

#### 2.4.2. ABTS^+^· Radical Scavenging Activity Assay

The ABTS^+^· radical scavenging assay was conducted according to published protocols [26]. Briefly, the ABTS^+^· working solution was prepared by mixing equal volumes of 7 mmol/L ABTS solution and 2.45 mmol/L potassium persulfate solution, followed by incubation in the dark at room temperature for 12–16 h. Prior to use, the solution was diluted with anhydrous ethanol to achieve an absorbance of 0.70 ± 0.02 at 734 nm.

TPS samples were prepared at gradient concentrations of 0.8, 2.0, 3.0, and 4.0 mg/mL. For each measurement, 100 μL of sample solution was mixed with 3 mL of the ABTS^+^· working solution. The mixture was incubated at room temperature for 6 min, and absorbance was measured at 734 nm (A_sample). A blank control (A_blank) was prepared by replacing the sample solution with 100 μL of ultrapure water. The ABTS^+^· radical scavenging rate was calculated as follows:

Scavenging rate (%) = [1 − (A_sample/A_blank)] × 100.(3)

### 2.5. Molecular Docking of VOCs with Olfactory Receptors

Molecular docking was performed to investigate the binding interactions between the key VOCs identified and six representative olfactory receptors (ORs). The six human olfactory receptors used in this study were OR1A1 (UniProt ID: Q9P1Q5), OR1G1 (UniProt ID: P47890), OR2W1 (UniProt ID: Q9Y3N9), OR5M3 (UniProt ID: Q8NGP4), OR7D4 (UniProt ID: Q8NG98), and OR8D1 (UniProt ID: Q8WZ84). Their amino acid sequences of the two ORs were retrieved from UniProt, and their three-dimensional (3D) models were generated by homology modeling using SWISS-MODEL. The models were refined via energy minimization, and their quality was verified by Ramachandran plot analysis along with QMEAN and GMQE scores. The 3D conformations of the VOCs were obtained from the PubChem database. Molecular docking was performed using AutoDock Vina 1.2.5. For OR1A1, the grid box was centered at (2.255, −2.478, 3.910) with dimensions of 37.5 × 37.5 × 37.5 Å. The exhaustiveness, num_modes, and energy range were set to 32, 9, and 4 kcal/mol, respectively.

### 2.6. Data Processing and Statistical Analysis

All experiments were performed in triplicate. One-way analysis of variance (ANOVA) followed by Duncan’s multiple range test (*p* < 0.05) was conducted using IBM SPSS Statistics 27 software. Multivariate statistical analysis of volatile metabolites was performed using SIMCA 14.1 software. Data were first subjected to unit variance scaling, followed by principal component analysis (PCA) to observe overall trends. Supervised partial least squares discriminant analysis (PLS-DA) was subsequently applied to maximize differentiation among cultivars, and variables with variable importance in projection (VIP) scores greater than 1.0 were selected as key differential volatile compounds. For TPS in vitro antioxidant activity, IC_50_ values were calculated by fitting a four-parameter logistic equation using GraphPad Prism 10.4.1 software, with sample concentration as the independent variable and radical scavenging rate as the dependent variable. Lower IC_50_ values indicate stronger antioxidant capacity. To standardize the antioxidant capacity across different samples, the IC_50_ value of ascorbic acid was used as a reference standard, and the results were also expressed as ascorbic acid equivalent antioxidant capacity (AEAC, mg VcE/g TPS), calculated as

AEAC = IC_50_ (Vc)/IC_50_ (TPS) × 1000. (4)

## 3. Results

### 3.1. Characterization of Volatile Compound Profiles in Six Anxi Oolong Tea Cultivars

In this study, HS-SPME-GC-MS was employed for untargeted analysis of VOCs (Figure 1B). A total of 39 volatile compounds were identified, of which 30 were detected in at least two cultivars, spanning major chemical classes including alcohols, aldehydes, ketones, and esters (Figure 1C). Additionally, 10 volatiles were detected in all six cultivars. Among these shared compounds, trans-nerolidol (CAS 40716-66-3), indole (CAS 120-72-9), and α-farnesene (CAS 502-61-4) exhibited relatively large peak areas (Figure 1B). Notably, dihydrolinalool (CAS 29957-43-5) showed the highest content in DYL, representing a unique feature of this cultivar.

Regarding quantified total volatile content, significant differences were observed among the six cultivars (Figure 1C). The high-aroma cultivar HJG possessed the highest total VOC content at 69.01 μg·g^−1^, demonstrating a marked advantage over other cultivars. Overall, alcohols constituted the largest proportion of total volatiles, while esters and lactones represented the smallest fraction (Figure 1C). HJG exhibited the highest alcohol content (32.31 ± 8.01 μg·g^−1^), significantly exceeding all other cultivars. In contrast, MX showed the lowest alcohol content at only 12.44 ± 2.24 μg·g^−1^. Furthermore, aldehydes, indole, and terpenes also accounted for substantial proportions across cultivars, contributing importantly to the overall aroma characteristics.

Principal component analysis further demonstrated significant differences in volatile metabolite profiles among cultivars. The PCA score plot revealed clear separation trends among the six oolong tea cultivars (Figure 1D), indicating substantial inter-cultivar variation in volatile composition. Hierarchical clustering analysis (HCA) corroborated this finding. Clustering results showed that BSS, HJG, and MX had relatively similar volatile profiles, while TGY and MZ clustered together (Figure 1E). The metabolome of DYL was notably more independent, exhibiting the greatest distance from the other five cultivars (Figure 1E). The consistency between PCA and HCA results established a foundation for distinguishing the volatile characteristics of the six cultivars.

### 3.2. Identification of Differential Volatiles Among Six Cultivars Based on Metabolomics

To further identify candidate markers for distinguishing cultivar-specific aroma metabolites, a supervised classification model was established using PLS-DA. The model demonstrated high explanatory power (R^2^Y = 0.979) and predictive ability (Figure 2A,B). With a VIP threshold of >1.0, 14 key differential volatiles were identified as candidate marker compounds for discriminating among cultivars (Figure 2C).

The actual aroma contributions of these differential volatiles were evaluated by calculating their relative odor activity values (rOAVs). A total of 28 compounds exhibited rOAV > 1 in at least one cultivar, indicating their potential as major contributors to cultivar-specific aroma characteristics. Notably, HJG contained 20 compounds with rOAV > 1, consistent with its recognition as a “high-aroma” cultivar and highlighting its complex and pronounced aroma profile. This aligns with practical production experience where HJG is widely regarded as possessing intense aromatic properties. Based on the combined VIP and rOAV analyses, 12 volatiles were further screened, including trans-nerolidol, indole, linalool (CAS 78-70-6), geraniol (CAS 106-24-1), and benzaldehyde (CAS 100-52-7). These metabolites exhibited both significant metabolic differentiation and rOAV values > 1, constituting the core active compounds responsible for inter-cultivar aroma differences (Figure 2D,E). Among these, trans-nerolidol, phenylacetaldehyde (CAS 122-78-1), indole, and α-farnesene exhibited relatively high rOAV values across all cultivars, contributing significantly to the overall aroma. Linalool also participated in aroma formation, with rOAV values over 0.1 (Figure 2D). Collectively, these five volatiles constitute the essential skeleton of the floral aroma characteristics shared among the six cultivars. The remaining seven volatiles showed substantial variation in content and aroma contribution across different cultivars (Figure 2E).

Overall, these 12 key metabolites collectively form the chemical basis for cultivar-specific aroma in oolong tea, with particularly pronounced contributions to floral characteristics. This supports the sensory attribute pattern of “floral aroma” dominance in Minnan oolong teas. Additionally, green-note volatiles were relatively more abundant in BSS and HJG, consistent with a possible regulatory influence of cultivar-specific genetic backgrounds on aroma metabolites, although direct genetic evidence is not provided in this study.

### 3.3. Molecular Docking Analysis of Differential Volatiles with Olfactory Receptors

To obtain preliminary in silico estimates of potential binding interactions between the 12 selected differential volatiles and human olfactory receptors, molecular docking was performed with OR1A1 and five other typical olfactory receptors. The preliminary in silico estimates indicated that all volatiles could form stable intermolecular interactions with all six tested olfactory receptors, with predicted binding energies ranging from −7.54 to −3.68 kcal/mol (Figure 3A). These estimates suggest possible receptor interactions and may help prioritize compounds for future sensory or receptor-function studies, but they are not direct evidence of olfactory perception. Among the tested volatiles, trans-nerolidol exhibited the strongest predicted affinity for OR1A1 (−7.54 kcal/mol), suggesting that it may be a relatively favorable ligand among the tested compounds. Such in silico predictions require experimental verification.

In the best docking pose of the nerolidol OR1A1 complex, a total of nine intermolecular interactions were predicted, comprising hydrophobic interactions and hydrogen bonds (Figure 3B). Among them, eight hydrophobic interactions were predicted, potentially facilitating the stable embedding of the ligand into the hydrophobic pocket. Further analysis of binding modes in silico suggested that hydrogen bonding and hydrophobic interactions were predicted to be the primary mechanisms potentially underlying volatile-olfactory receptor binding. On average, approximately 2.7 hydrogen bonds were predicted to form per docked complex. Additionally, hot spot residue analysis suggested PHE206, VAL203, and VAL254 of OR1A1 as potential high-frequency hydrophobic binding sites. These amino acid residues may collectively form hydrophobic pockets that could enhance the binding stability of volatile compounds. These in silico data suggest that the identified volatiles may bind to olfactory receptors. Whether such binding contributes to the sensory neural perception of aroma differences requires confirmation by sensory evaluation.

### 3.4. Detailed Characterization of Cultivar-Specific Volatiles

Further analysis focused on elucidating the unique metabolic compounds within each of the six oolong tea cultivars. The results indicated that, overall, Qingxiang oolong tea was characterized by the prominent contribution of floral and green-note volatiles, while the specific aroma characteristics of each cultivar were dominated by a small number of unique compounds (Figure 4). Specifically, in TGY, (E)-2-octenal (CAS 2548-87-0) and 1-phenyl-2-nitroethane (CAS 6125-24-2) were exclusively detected, with both compounds exhibiting rOAV values exceeding 100. The absence of these compounds in other cultivars suggests their critical role in forming the unique “Yinyun” aroma characteristic. For MX, the specific compounds were 2-phenylethyl-2-methylbutyrate (CAS 24817-51-4) and cis-3-hexenyl isovalerate (CAS 53398-86-0). The estimated rOAV for the former exceeded 1, while the latter exhibited a high rOAV of 69, underscoring its importance in MX’s aroma contribution. Structural analogs of 2-phenylethyl-2-methylbutyrate, such as ethyl phenylacetate (CAS 101-97-3) and ethyl 2-methylbutyrate (CAS 7452-79-1), have reported thresholds in the range of 50–100 μg/kg, supporting the estimation that its rOAV exceeds 1 and contributes to MX’s aroma profile. In DYL, α-ocimene and cis-3-hexenyl benzoate showed notable specificity, both possessing floral characteristics, suggesting these two compounds are key contributors to DYL’s unique floral aroma. In MZ, (E,E,Z)-1,3,5,8-undecatetraene (CAS 50277-31-1) was found to be specifically present, indicating its specificity in MZ’s aroma perception. These cultivar-specific metabolites may serve as potential chemical markers for cultivar identification and quality monitoring.

### 3.5. Comparison of Tea Polysaccharide Content Among Six Oolong Tea Cultivars

To further investigate quality differences among the six famous Anxi teas under identical processing conditions, crude TPS content was determined. The results showed significant differences in TPS content among the six oolong tea cultivars (Figure 5), with levels ranging from 2.76% to 3.99%. TGY exhibited the highest TPS content at (3.99 ± 0.09)%, significantly surpassing all other cultivars. MZ showed the lowest TPS content at (2.76 ± 0.05)%. The TPS content of TGY was 1.45 times that of MZ. Statistical analysis revealed that the TPS contents of TGY, DYL, and HJG were significantly different from those of the other three cultivars (MX, BSS, and MZ) (*p* < 0.01). However, differences among MX, BSS, and MZ did not reach statistical significance, suggesting potentially more similar metabolic characteristics among these three cultivars. As a critical component influencing the antioxidant and functional properties of oolong tea, crude TPS content differed significantly among cultivars, suggesting a possible genetic contribution to polysaccharide metabolism that warrants direct validation in future studies.

### 3.6. Comparison of Free Radical Scavenging Capacity of Tea Polysaccharides from Oolong Tea

To explore the in vitro antioxidant performance of TPS from different cultivars, DPPH radical scavenging capacity was assessed. The results demonstrated that the DPPH radical scavenging rates of TPS from all six cultivars increased with increasing polysaccharide concentration. Using half-maximal inhibitory concentration (IC_50_) as an indicator, significant differences in DPPH radical scavenging capacity were observed among cultivars (Figure 6A). TPS from DYL exhibited the lowest IC_50_ value (0.58 mg·mL^−1^), indicating the strongest scavenging ability. In contrast, TPS from MZ showed the highest IC_50_ value (1.69 mg·mL^−1^), reflecting the weakest scavenging capacity. The in vitro DPPH radical scavenging capacity of DYL TPS was significantly higher than that of TGY, HJG, and the other three cultivars (*p* < 0.05). We hypothesize that this enhanced activity may be attributed to a higher proportion of active hydroxyl groups or other structural features favorable for radical scavenging. Since the TPS fractions were crude and their structural parameters were not experimentally determined, direct structural characterization is required to confirm this hypothesis. When compared with vitamin C as a positive control, the DPPH scavenging efficiency of DYL TPS, although substantial, remained lower than that of vitamin C at equivalent mass. To enable direct comparison with the positive control, the DPPH scavenging capacity was further expressed as AEAC, which normalized the inhibition data against the IC_50_ of ascorbic acid. The AEAC values for DPPH scavenging ranged from 124.3 to 362.1 mg VcE/g TPS, with DYL exhibiting the highest AEAC (362.1 mg VcE/g TPS) and MZ the lowest (124.3 mg VcE/g TPS). This cultivar ranking of radical scavenging capacity was fully consistent with that based on IC_50_ values.

The ABTS^+^· radical scavenging capacity of TPS was further evaluated (Figure 6B). Consistent with the DPPH assay results, the ABTS^+^· scavenging rates of TPS from all six cultivars increased with concentration. Comparison of IC_50_ values for ABTS^+^· scavenging revealed significant differences among cultivars (Figure 6B). TPS from DYL exhibited the lowest IC_50_ value (1.95 mg·mL^−1^) and the highest AEAC (117.9 mg VcE/g TPS), demonstrating the strongest scavenging capacity. Meanwhile, TPS from MZ showed the highest IC_50_ value (3.33 mg·mL^−1^) and the lowest AEAC (69.1 mg VcE/g TPS), indicating the weakest chemical antioxidant capacity. In agreement with the DPPH results, the AEAC values for ABTS^+^· scavenging ranged from 69.1 to 117.9 mg VcE/g TPS. This cultivar ranking mirrored the ranking derived from IC_50_ values. Overall, these cultivar differences are hypothesized to reflect differences in TPS structure and composition, but confirmation requires structural characterization. DYL demonstrated superior antioxidant performance in this study, performing well in both DPPH and ABTS^+^· radical scavenging assays. Notably, while TGY possessed the highest TPS content, its in vitro antioxidant activity was lower than that of DYL, suggesting, as a hypothesis, that beyond polysaccharide content, other factors such as protein content, polyphenol content, and glycosidic linkage types may substantially influence chemical antioxidant activity. Since these parameters were not experimentally determined in this study, this explanation remains a testable hypothesis that warrants direct validation.

## 4. Discussion

Genomic studies have revealed that the terpene synthase gene family in high-aroma cultivars such as HJG possesses significantly enriched structural variations and specific high expression, establishing a genetic basis for the differential accumulation of terpenoid aroma compounds [13,14,15]. Under strictly controlled conditions of identical habitat and uniform Qingxiang oolong tea processing, the six Anxi oolong tea cultivars exhibited clear separation in volatile metabolic profiles, consistent with these genomic findings and strongly suggesting that cultivar genetic background plays a major role in shaping aroma differences. Terpene synthases have been confirmed to catalyze the synthesis of diverse terpenoid products, including linalool, geraniol, limonene, and ocimene [27,28,29]. The key metabolites identified in this study include numerous terpenoid volatiles, such as trans-nerolidol, linalool, and α-farnesene. The high-aroma characteristic of HJG was suggested, as it exhibited the highest total VOC content (69.01 μg·g^−1^) and the greatest number of aroma-contributing compounds (20 compounds with rOAV > 1). Previous research has also found that (E)-2-octenal content in TGY is higher than in other cultivars [30]. Our results are consistent with the identification of core odorants in Fujian oolong teas [9]. Multiple metabolomic studies have demonstrated clear differentiation of terpenoid aroma substances among cultivars [31,32,33]. Among the 12 key differential metabolites, six were terpenoids. This supports the idea that terpenoids contribute to the aroma differences among oolong tea cultivars. The preliminary in silico docking estimates predicted that hydrogen bonding and hydrophobic interactions contribute importantly to VOCs binding to olfactory receptors, including OR1A1 and five typical human olfactory receptors, consistent with previous findings on floral-aroma tea [34].

Regarding TPS differences, TGY exhibited the highest crude TPS content (3.99%), which was 1.45 times that of MZ (2.76%). TGY, DYL, and HJG all had crude TPS contents exceeding 3.0%. In terms of chemical antioxidant activity, crude TPS from DYL demonstrated the strongest DPPH and ABTS^+^· scavenging capacities (IC_50_ = 0.58 and 1.95 mg·mL^−1^, respectively), while MZ showed the weakest scavenging activity (IC_50_ = 1.69 and 3.33 mg·mL^−1^, respectively). Notably, although TGY had the highest polysaccharide content, its in vitro chemical radical scavenging capacity was significantly lower than that of DYL. These findings indicated that the radical scavenging capacity of crude TPS differed among cultivars. However, these capacities were measured in cell-free chemical systems and therefore do not necessarily represent in vivo antioxidant activity [35,36]. In addition, because the structural parameters of TPS were not characterized in this study, whether these differences arise from structural features remains to be validated. Nevertheless, previous studies have shown that the biological activity of TPS is directly related to structural features such as monosaccharide composition, molecular weight, glycosidic linkages, configuration, and conformation [5,37]. Previous studies have found that the radical scavenging capacity of TPS from different tea cultivars is correlated with their polyphenol and protein content [38]. Comparative studies focusing on structural characterization have demonstrated that the biological activity of polysaccharides is significantly correlated with their protein and uronic acid content [19]. Moreover, removing metal ions from TPS significantly improves the in vitro chemical radical scavenging capacity of neutral polysaccharide fractions [26]. Earlier research also found that the degree of fermentation during processing significantly affects the protein, total sugar, and uronic acid content of TPS, and the same studies reported higher in vitro chemical radical scavenging capacity for fully fermented dark teas [39,40]. In line with these observations, studies on multiple tea germplasms and crude TPS fractions have reported significant inter-cultivar variation in uronic acid, protein, polyphenol, and monosaccharide composition and have shown that these structural parameters, together with molecular weight and glycosidic linkages, are associated with in vitro radical scavenging activity [20]. The present study was conducted under identical habitat and processing conditions. Under these conditions, we found that crude TPS from DYL possessed superior chemical antioxidant activity compared to those from TGY. In light of the above evidence, it is hypothesized that the high in vitro radical scavenging capacity of TPS in DYL may stem from structural features such as a higher proportion of active hydroxyl groups, uronic acid content, or protein content. However, because the TPS fractions were crude and these structural parameters were not directly measured in this study, this interpretation remains a hypothesis and requires direct structural and mechanistic validation.

## 5. Conclusions

This study systematically revealed cultivar differences in volatile metabolite profiles and TPS activity among six oolong tea cultivars under identical Qingxiang processing conditions. The chemical composition and functional activity still exhibited significant differentiation among cultivars, consistent with cultivar genetic background being a potentially key intrinsic factor determining tea quality characteristics. A total of 12 key differential volatiles were identified as the chemical basis of floral aroma. Notably, crude TPS from DYL showed the strongest in vitro chemical radical scavenging capacity despite not having the highest polysaccharide content. This results tentatively suggests that structural features, rather than content alone, may govern the scavenging capacity of TPS, but direct structural characterization is required to confirm this. These findings provide a theoretical basis for cultivar selection and quality optimization in oolong tea production. However, the present study only measured polysaccharide content and in vitro chemical radical scavenging capacity, without systematic characterization of molecular weight distribution, monosaccharide composition, uronic acid content, or bound protein and polyphenol content. Future research should incorporate monosaccharide composition analysis, uronic acid and protein quantification to further elucidate the structure-activity relationships between the fine structure of TPS and their in vitro antioxidant activity, thereby providing a more robust theoretical foundation for cultivar-specific quality regulation during tea processing.

## Figures and Tables

**Figure 1:**
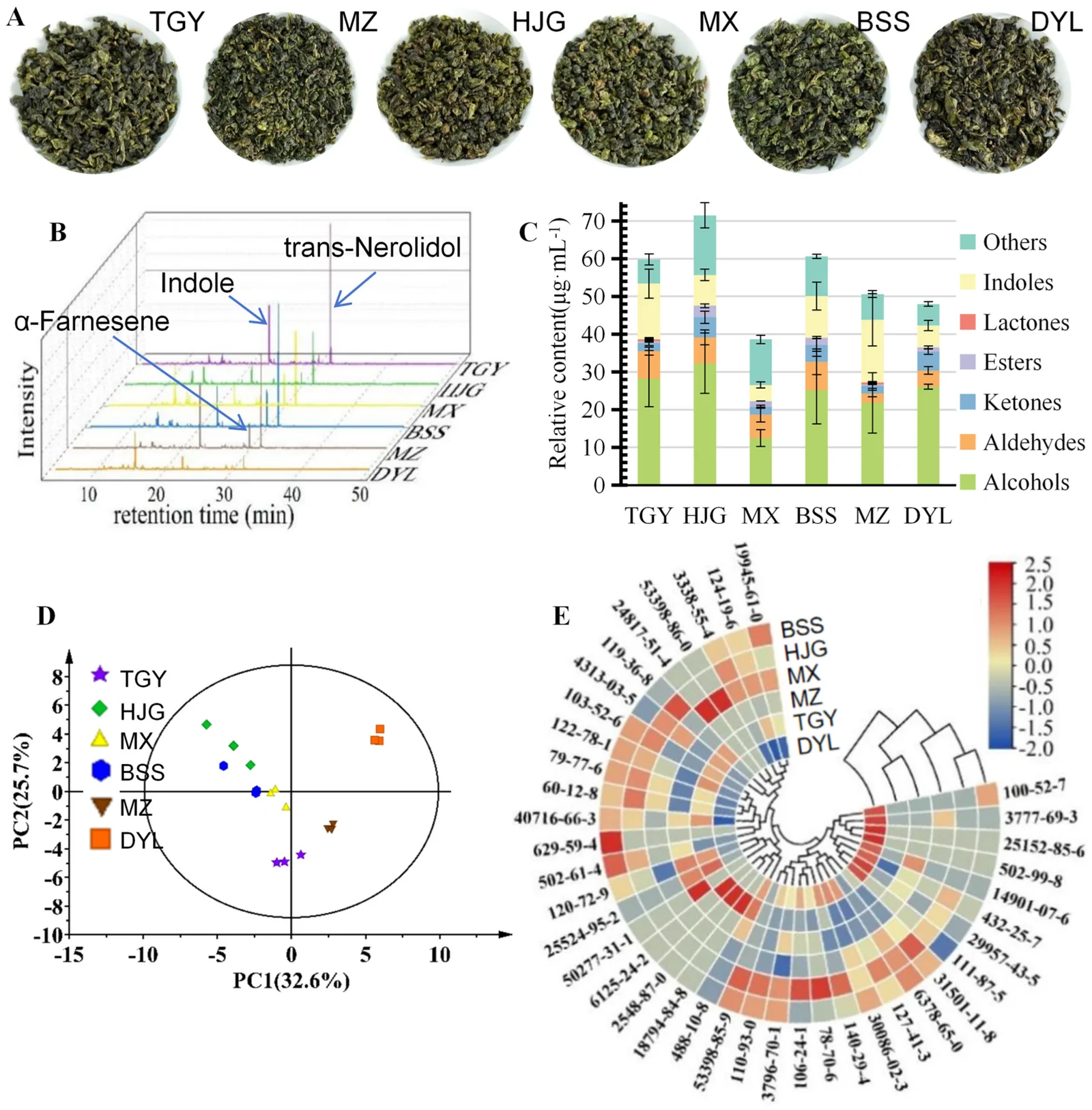
Volatile compound profiles in six Anxi oolong tea cultivars. (**A**) Qingxiang oolong tea samples of six cultivars prepared under identical conditions. (**B**) Overview of volatile compound profiles across the six cultivars. (**C**) Distribution of volatile compounds by chemical class. (**D**) Principal component analysis score plot of volatile profiles from the six cultivars. (**E**) Hierarchical clustering analysis dendrogram of volatile profiles from the six cultivars. Abbreviations: TGY, Tieguanyin; HJG, Huangjingui; MX, Maoxie; BSS, Benshan; MZ, Meizhan; DYL, Daye Wulong.

**Figure 2:**
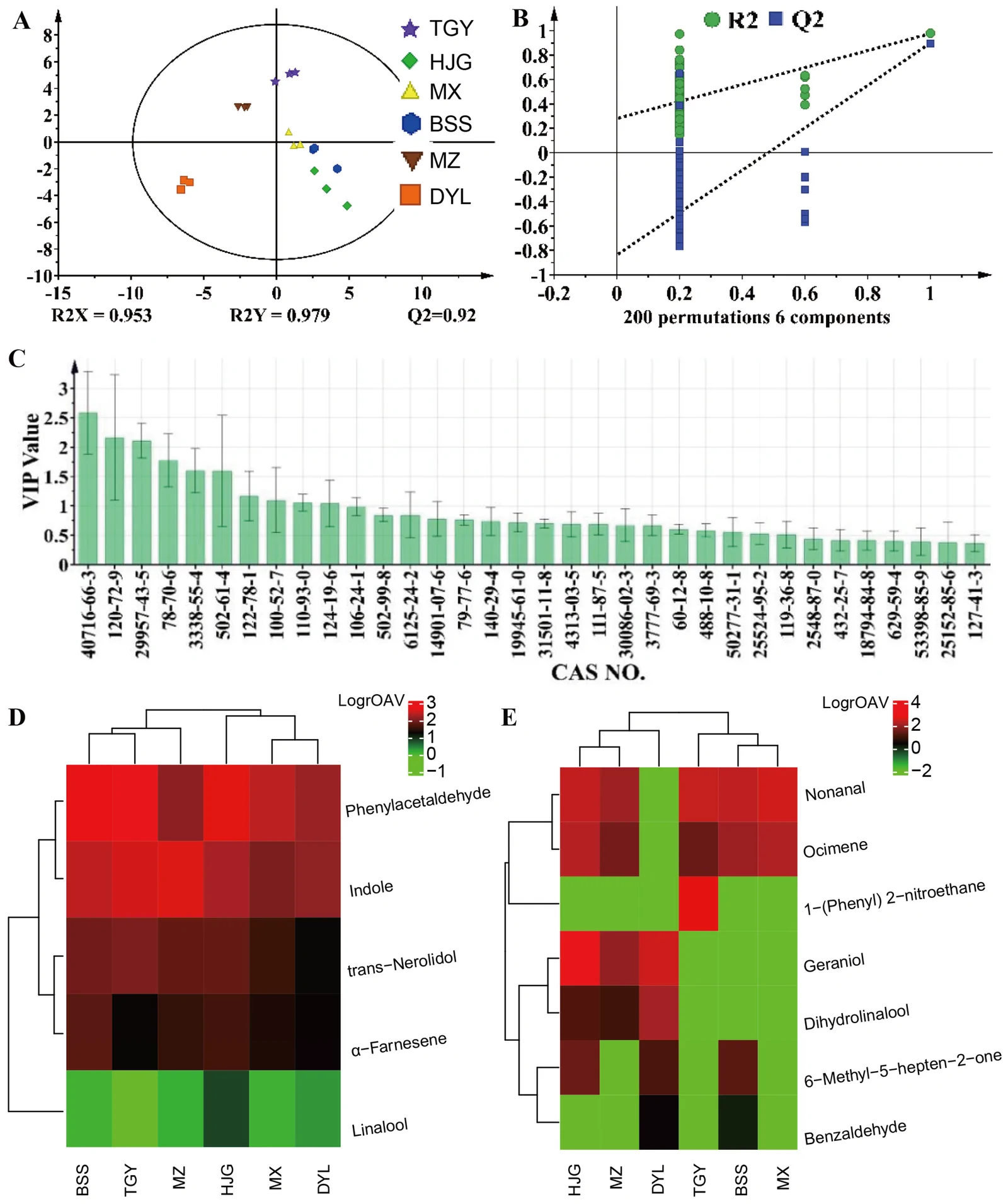
Key differential volatiles among six Anxi oolong tea cultivars. (**A**) PLS-DA score plot of volatile profiles from six Anxi oolong tea cultivars. (**B**) Permutation test for PLS-DA model used to differentiate the six Anxi oolong tea cultivars. (**C**) VIP scores of volatiles. (**D**) Heatmap of key differential volatiles constituting the floral aroma skeleton. (**E**) Heatmap of cultivar-specific key differential volatiles. Abbreviations: TGY, Tieguanyin; HJG, Huangjingui; MX, Maoxie; BSS, Benshan; MZ, Meizhan; DYL, Daye Wulong.

**Figure 3:**
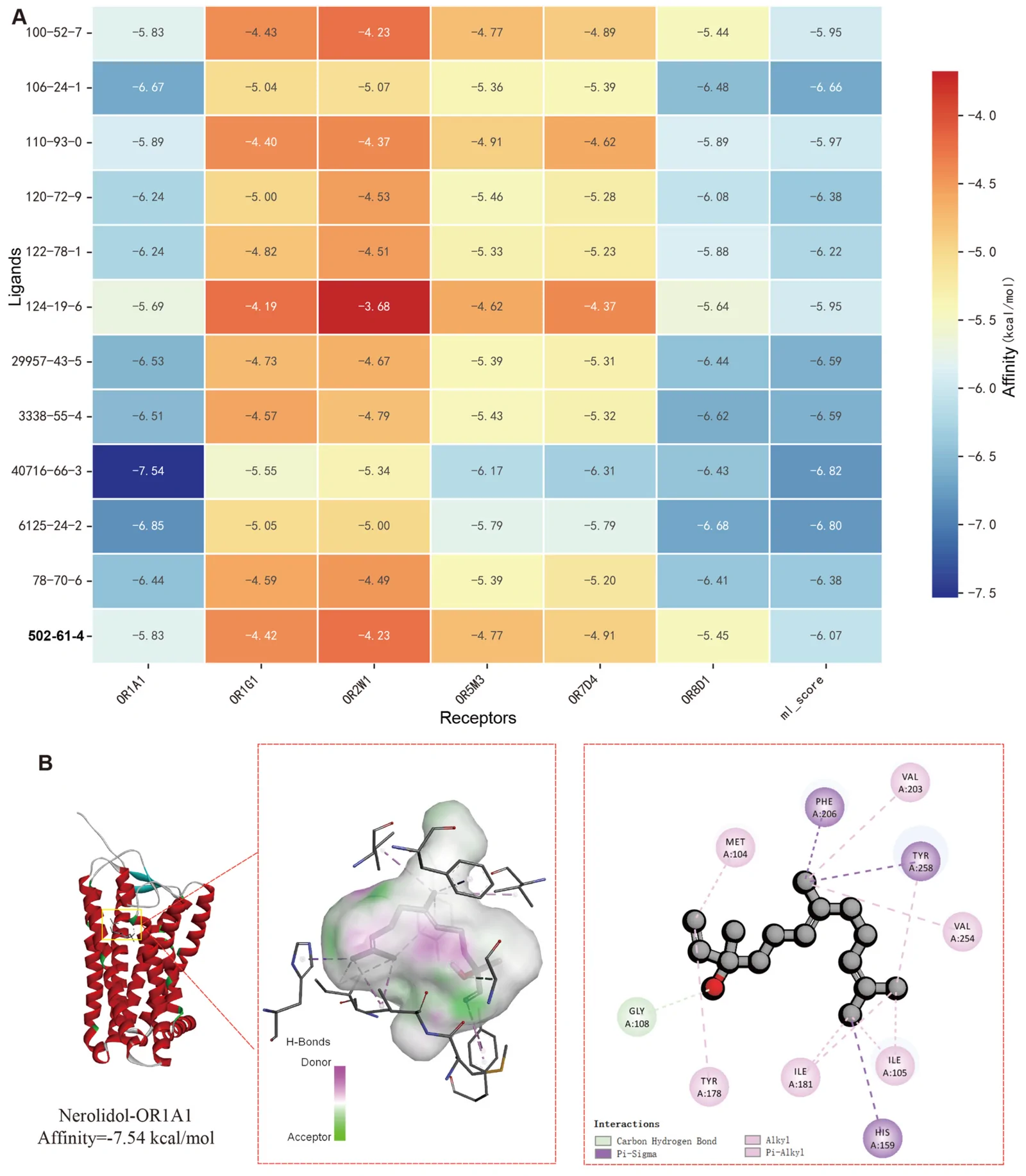
Preliminary in silico docking analysis of key differential volatiles with olfactory receptors. (**A**) Heatmap of predicted binding energies between 12 key volatiles and six typical olfactory receptors. (**B**) Best docking pose of the nerolidol OR1A1 complex. Spheres are colored by atom type: gray, carbon; red, oxygen.

**Figure 4:**
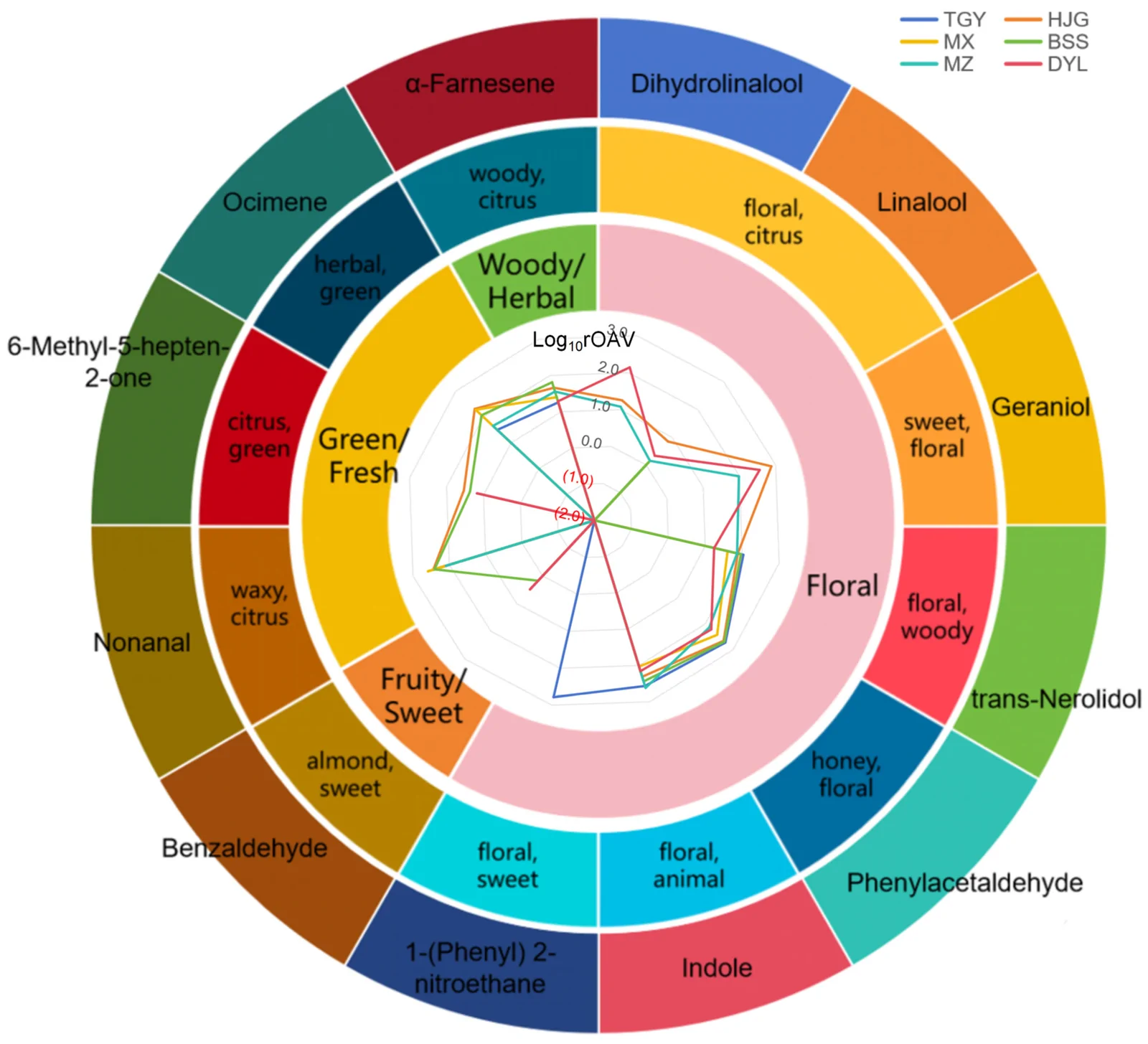
Flavor wheel and rOAV radar chart of cultivar-specific volatiles in six Anxi oolong tea cultivars. Abbreviations: TGY, Tieguanyin; HJG, Huangjingui; MX, Maoxie; BSS, Benshan; MZ, Meizhan; DYL, Daye Wulong.

**Figure 5:**
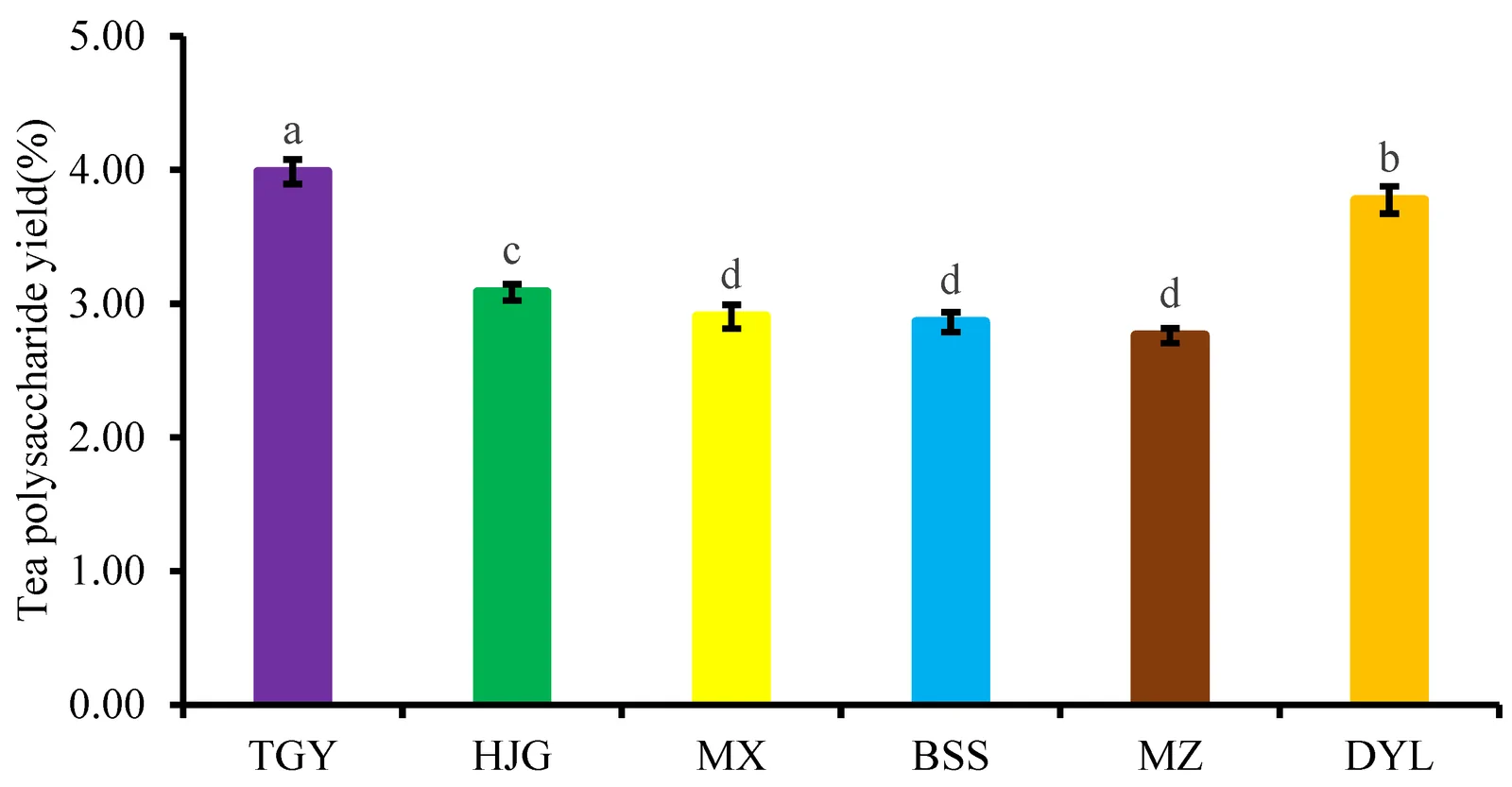
Crude tea polysaccharide content from six Anxi oolong tea cultivars. Abbreviations: TGY, Tieguanyin; HJG, Huangjingui; MX, Maoxie; BSS, Benshan; MZ, Meizhan; DYL, Daye Wulong. Different lowercase letters indicate significant differences among cultivars (*p* < 0.05).

**Figure 6:**
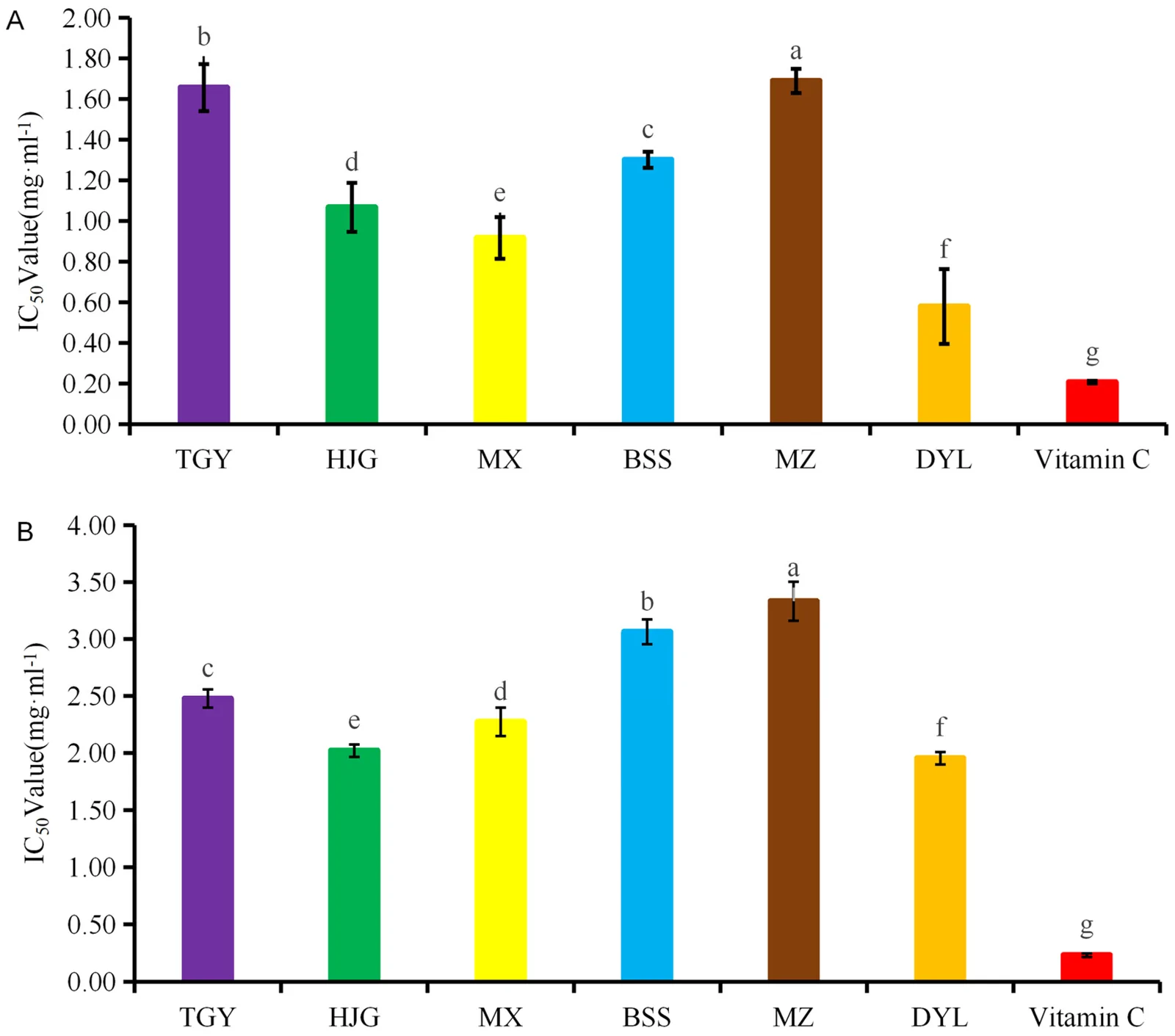
In vitro chemical radical scavenging capacities of crude tea polysaccharides from six Anxi oolong tea cultivars. (**A**) DPPH radical scavenging capacity. (**B**) ABTS^+^· radical scavenging capacity. Abbreviations: TGY, Tieguanyin; HJG, Huangjingui; MX, Maoxie; BSS, Benshan; MZ, Meizhan; DYL, Daye Wulong. Different lowercase letters indicate significant differences among cultivars (*p* < 0.05).

## Data Availability

The original contributions presented in this study are included in the article. Further inquiries can be directed to the corresponding author.
